# Cognitive Style Shaped by Framing: Implications for Rational vs. Intuitive Thinking

**DOI:** 10.3390/bs16050823

**Published:** 2026-05-19

**Authors:** Marcus Selart

**Affiliations:** Department of Strategy and Management, Norwegian School of Economics, 5045 Bergen, Norway; marcus.selart@nhh.no

**Keywords:** cognitive style, personality, holistic dependence/independence on framing, analytical intelligence, rationality, intuition

## Abstract

This article investigates how cognitive style shaped by framing influences rational and intuitive thinking. We extend existing research on cognitive styles, specifically the construct of holistic dependence/independence on framing, and examine its relationship with reasoning performance, self-rated intuition, and analytical intelligence. We conduct tests with university students and find that individuals with high holistic independence on framing perform better on rationality tests and are better predictors of rational thinking than traditional measures of intelligence. Additionally, holistic independence on framing was found to be marginally related to lower self-rated reliance on intuition. These findings suggest that holistic dependence/independence on framing may provide additional insight into reasoning styles beyond traditional intelligence measures, highlighting its potential relevance for understanding individual differences in decision-making processes.

## 1. Introduction

The issue of cognitive styles has been highly discussed in the research literature for a long time, since they constitute preferred ways of processing information as well as well-functioning predictors in educational and occupational settings. Scientists have studied these styles from cognition-centred, personality-centred, and activity-centred perspectives. It has been proposed that cognitive styles are continuous, socially influenced, and functionally related to how individuals manage their thinking processes (Sternberg & Grigorenko, 1997). Cognitive styles have successfully offered a valuable complement to ability measures.

The central idea of the present study is to focus on cognitive style shaped by framing and its implications for thinking processes. In this mission, we build on the older research tradition that has studied the cognitive style formerly known as field dependence/independence. Here, it is assumed that the extent to which individuals are susceptible to figure-embedded framing has a bearing on their holistic dependence/independence on framing in other more specialized fields, such as reasoning in judgment and decision making tasks. Such framing is often based on the changing of reference points or by manipulating outcome salience and response mode (Tversky & Kahneman, 1981; Kahneman & Tversky, 1983; Tversky & Kahneman, 1986; Entman, 1993; Nelson et al., 1997; Kuhberger, 1998; Levin et al., 1998; Kuvaas & Selart, 2004; Chong & Druckman, 2007; Aarøen & Selart, 2025). Individuals with low holistic independence on framing are more influenced by the surrounding context and may find it difficult to identify embedded figures within complex backgrounds. In contrast, those with high holistic independence are less affected by contextual distractions, allowing them to more easily disregard irrelevant information. As a result, they tend to have shorter response times and are able to detect more embedded figures (Witkin, 1973; Witkin et al., 1977).

Holistic dependence/independence on framing is conceptualized as a bipolar, one-dimensional model of cognitive style that describes how individuals perceive and process information. Specifically, it refers to the ability to distinguish object figures from the distracting or confusing background, known as the content field (Witkin, 1973). Instruments measuring this construct are designed to classify individuals as either field-independent or field-dependent, with the classification intended to be value-neutral. Field-independent (holistic independent) individuals are generally more autonomous in developing restructuring skills required for unfamiliar technical tasks, whereas they may be less autonomous in developing interpersonal skills (Witkin et al., 1977).

This form of cognitive style may be denoted as peoples’ dependence or independence from their perceptual field and how it impacts their organization of impressions (Oltman et al., 1975; Goodenough, 1976; Witkin & Goodenough, 1977, 1981). It is often referred to as analytical vs. holistic cognitive style (Riding & Cheema, 1991; Allinson & Hayes, 1996; Kozhevnikov, 2007) but terms like psychological differentiation and closure flexibility can also be found in the literature. We proffer the construct holistic dependence/independence on framing based on a call from research in which the relationship between cognitive style and framing is presented in many different ways (Levin et al., 2002; Keller et al., 2003; Gross & D’Ambrosio, 2004; Lauriola et al., 2005; Druckman & McDermott, 2008; Gross, 2008; Covey, 2014; Giorgi, 2017). The proposed construct addresses consistent individual differences with regard to preferred ways of organizing and processing information and experiences (Messick, 1984). Thus, research suggests that this form of cognitive style bridges two important subfields within psychology namely cognition and personality (see also Myers, 1962; Witkin et al., 1977; Riding & Cheema, 1991; Riding & Sadler-Smith, 1992; Hayes & Allinson, 1994; Allinson & Hayes, 1996; Riding, 1997; Sternberg & Grigorenko, 1997; Hayes & Allinson, 1998).

A cognitive style refers to an individual’s habitual, consistent, and preferred way of thinking, perceiving, remembering, and problem-solving, acting as a link between personality and cognition. It is primarily identified through observed individual differences in cognitive and perceptual functioning (Grigorenko & Sternberg, 1995; Sternberg & Grigorenko, 1997; Bendall et al., 2016). Most of the assessment methods depend on self-report measures, and this fact has been subject to recent critique, (Rayner & Riding, 1997; Coffield et al., 2004; Cools, 2009; Bendall et al., 2016) such that style design that uses multiple methods are believed to offer greater potential for validation (Spratt et al., 2004; Cassidy, 2012). Nevertheless, this critique has to a large extent been neglected by cognitive styles research. Although cognitive styles are generally considered stable over time, some researchers argue that they may be more dynamic and subject to change (Zhang, 2013). Still, the prevailing consensus supports the view that cognitive styles exhibit long-term stability and are resistant to modification (Clapp, 1993; Riding & Rayner, 1998; Sadler-Smith et al., 2000; Sternberg & Zhang, 2001; Kirton, 2003; Zhang & Sternberg, 2006; Cools & Van den Broeck, 2007; Cools et al., 2009; Zhang & Sternberg, 2009; Armstrong et al., 2012; Cools et al., 2014).

### 1.1. The Impact of Cognitive Style on Analytical and Intuitive Decision Processes

In this study we will investigate how holistic dependence/independence on framing influences peoples’ performance on analytical reasoning tasks and preference for either analytic or intuitive decision styles. We will also investigate whether holistic dependence/independence on framing has discriminative validity in relation to analytical intelligence. Our aim is to introduce holistic dependence/independence on framing as an important predictor of reasoning skills and decision making (see e.g., Stanovich & West, 1998, 2000). People with low holistic independence on framing are considered to be more social, group-oriented, and socially competent than people with high such independence (Witkin & Goodenough, 1981; Witkin et al., 1977). They are also assumed to be more sensitive to social interaction and critique, more externally motivated, and to a higher extent dependent on external reference points in managing their social life. People with high holistic independence on framing, on the other hand, are often described as more internal in their locus of control, more internally motivated, and more analytically task focused (Fritz, 1994; Liu & Reed, 1994; Lyons-Lawrence, 1994; Reiff, 1996; Riding & Cheema, 1991; Glambek et al., 2026). Furthermore, individuals with low holistic independence on framing seem to remember more of the surrounding context of an event whereas individuals that are scoring high on this kind of independence are much more attentive to details (Eagle et al., 1969). Evidence from recent studies suggests that holistic dependence/independence on framing correlates with intelligence, working memory, and verbal/spatial abilities (Jones, 1997; Linn & Kyllonen, 1981; López-Rupérez et al., 1991; MacLeod et al., 1986; McKenna, 1984; Miyake et al., 2001; Moutier et al., 2002; Tsaparlis, 2005).

Focusing on different thinking styles, Allinson and Hayes (1996) differentiate between analytic thinking, which implies processing information in an ordered, linear sequence, and holistic thinking, which involves viewing the whole situation at once in order to facilitate the synthesis of all available information. These modes of cognition are labelled “analytic” and “intuitive” respectively. The scope of Pennycook et al. (2015) has been to examine the everyday consequences of analytical thinking, focusing on how individuals’ propensity to engage in reflective reasoning influence their beliefs, moral judgments, and social behaviors. They review evidence showing that higher analytic thinking is associated with greater scepticism toward different forms of beliefs as well as with less emotional and more rational judgments (see also Damasio, 2005; Newell, 2015; Evans, 2008; Stanovich et al., 2011; Toplak et al., 2011; Mercier & Sperber, 2011; Evans & Stanovich, 2013). They also find that analytic thinkers tend to be less cooperative in social dilemmas but may exhibit greater creativity and less reliance on technology for offloading thinking (see also Brown et al., 2021; Fiske et al., 2007; Moore et al., 2011). These findings indicate that individual differences in analytic thinking significantly affect various psychological domains, broadening our understanding of how reflective reasoning shapes cognition and social attitudes. This perspective extends beyond previous research, which primarily emphasized cognitive ability (see also Strack & Deutsch, 2004; Beaty et al., 2014; Rand et al., 2012, 2014; Campitelli & Gerrans, 2014; Garvey & Ford, 2014).

Based on this reasoning, we make the following two hypotheses:

**H1:**  *Individuals with high holistic independence on framing will be able to think more rational than individuals that score low on this type of resistance.*

**H2:**  *Individuals with low holistic independence on framing will report higher levels of intuitive thinking than individuals that score high on this type of resistance. This suggests that individuals with low holistic independence on framing may have greater difficulty engaging in the controlled cognitive processes required for analytical decision making, and therefore are expected to perform less well on such tasks compared to those with high holistic independence.*

### 1.2. The Impact of Cognitive Style vs. Cognitive Ability on Analytical and Intuitive Decision Processes

Cognitive style and intelligence are generally considered distinct, independent constructs, where intelligence refers to the *level* of cognitive ability (how well one performs), and cognitive style refers to the *manner* of processing information (how one prefers to think). While intelligence (IQ) improves performance across tasks, cognitive style is value differentiated, meaning different styles are better suited for specific tasks (Riding & Pearson, 1994; Sternberg & Grigorenko, 1997). Intelligence has shown to be a strong and consistent predictor of positive organizational outcomes such as leadership effectiveness (Schmidt & Hunter, 1998; Judge et al., 2004; Furnham & Horne, 2021; Schmidt & Oh, 2023; Glambek et al., 2026). It has been defined as an ability to understand complex ideas, to adapt effectively the environment, to learn from experience, and to engage in various forms of reasoning (Neisser et al., 1996). Research indicates that cognitive styles are largely independent of intelligence. A person can have a high or low IQ regardless of their cognitive style. Ability (intelligence) determines the maximum level of performance, while cognitive style denotes the approach to the task (Riding & Pearson, 1994; Sternberg & Grigorenko, 1997). A high-IQ individual may use a specific style, but that style itself does not dictate their intelligence level. Abilities are “value directional” (more is better), whereas cognitive styles are value differentiated (no single style is inherently superior).

Cognitive styles are considered a mediator between cognition and personality, focusing on consistent individual differences in organizing and controlling behaviour. It is important to note that some cognitive styles, such as holistic dependence/independence of framing, reveal minor correlations with specific types of intelligence tests (Sternberg & Grigorenko, 1997). Hence, these kinds of cognitive styles show promise for understanding some of the variation in performance that can not be accounted for by individual differences in abilities. They predict performance significantly and add to the prediction provided by ability tests (Riding & Pearson, 1994; Sternberg & Grigorenko, 1997). Stanovich and West (1998) have found that individual difference with regard to cognitive ability is greatest at a lower algorithmic level. However, individual differences concerning thinking dispositions, at a rational level, are informed by availability to a higher degree. This concept addresses the simplicity by which impressions produced by the situation are perceived. Based on this reasoning, it seems plausible to assume that holistic independence on framing may better explain differences in both *rational* and *intuitive* ability.

Thus, we propose the following:

**H3:**  *Holistic independence on framing is expected to explain differences in rational and intuitive thinking better than analytical intelligence.*

## 2. Method

### 2.1. Participants

The study was based on responses obtained from 72 students at a Scandinavian university who participated voluntarily and without any payment. Participants’ ages ranged from 19–40 years (*M* = 23.5; *SD* = 4.16). Nearly as many men as women took part in the study, that is, 48.6% were male and 51.4% were female. Only two participants did not complete the experiment.

### 2.2. Material

The present study is based on four tests that measure holistic independence/dependence on framing, analytical intelligence, self-rated intuition, and rationality. Participants’ were also asked about their age and gender.

### 2.3. Part I—Holistic Dependence/Independence on Framing

We applied the Gottschaldt test in order to measure holistic independence on framing in line with Witkin’s development of his Embedded Figures Test. The Gottschaldt test is a paper and pencil test where a participant’s task is to find a simple geometric figure in a distractingly complex pattern. This is followed by being asked to mark the figure when it is found. A broad range of such tasks were applied in the test and participants marked as many figures as they were able to perceived during a specified time limit (2 min per sub-task). Depending on the performance, participants received a variety of scores for the correctly marked figures. They could maximally obtain 47 points. According to Witkin et al. (1977) to be classified as holistic dependent, participants should lie half a standard deviation below the mean, whereas they should be half a standard deviation above the mean to be classified as holistic independent (*M* = 27.4, *SD* = 8.10, *N* = 72). Witkin used response latency time per task as his measure of holistic dependence-independence performance. Instead, we chose to distribute scores depending on the amount of correctly marked figures perceived within a time limit. The aim was to differentiate participants’ thinking styles in this connection, since we did not have the time and resources to test each participant with Witkin’s method. The internal consistency was high (*α* = 0.84).

### 2.4. Part 2—Analytical Intelligence

The test we applied in this context is a less advanced and less extensive version of Raven’s Advanced Progressive Matrices. It has been used by Mensa Sweden and we had their chairman’s approval to use it. Broadly speaking, Raven’s matrices are assumed to be reliable in the measurement of analytical intelligence (Carpenter et al., 1990). Thus we found an internal consistency of 0.75 using the split-half technique on a pilot group of 40 students. In addition we observed a test-retest reliability of 0.79 when we retested the material one week later on the same group. These results are very much in line with the reliability results from the Standard Progressive Matrices (SPM). With regard to validity, we have been informed by the chairman that the test should correlate between 0.75–0.82 with WISC-R scores obtained from a group of students.

The matrices are designed to measure the cognitive processes that, from a theoretical point of view, are assumed to drive general intelligence (Mackintosh, 1998). Recently, Stanovich and West (1998) used this test in order to measure analytical intelligence in their studies on rational thinking. The test that we applied included 18 sub-tasks, and participants could therefore maximally obtain 18 points on the test (*M* = 9.63, *SD* = 4.35, *N* = 72). A time limit of 10 min for the entire test was applied. The internal consistency was high (*α* = 0.90).

### 2.5. Part 3—Intuitive Thinking

The test used in this context was inspired by a test developed by Adelbratt (2004) measuring self-rated intuition in decision situations. It consisted of four underlying cognitive processes: a. associative intuition, b. matching intuition, c. accumulative intuition, and d. constructive intuition. In our test, participants were introduced to a series of everyday decision situations related to personal relationships, education, job alternatives, personal finances etc. The participants’ task is to indicate to what degree they used their intuition in the presented six situations. A Likert Scale with seven levels was used to measure responses. The lowest degree of intuition (Not at all) rendered 1 point whereas the highest degree (Exclusively) rendered 7 points. This implied a maximum score of 42 points (*M* = 27.8, *SD* = 3.9, *N* = 50). The internal consistency was in the lower range of what may be regarded as acceptable (*α* = 0.71).

### 2.6. Part 4—Analytical Thinking

The rationality test consists of two separate parts, focusing on syllogisms and statistical reasoning. The syllogism tasks should be solved by taking into account deductive reasoning. These tasks include two premises and one conclusion. Premises and conclusions are not always obtained from the real world to test the ability to think logically (ex. premises: P1 All leaders are managers, P2 Mahatma Gandhi was a leader, Conclusion: Mahatma Gandhi was a manager). Hence, the goal was to judge whether or not the conclusion was logically correct or incorrect on the basis of the premises, without being influenced by the real world appearance of the latter. The syllogism test was based on a study presented by Markovits and Nantel (1989) that also served as inspiration for Stanovich and West (1998). Each correct answer rendered a score of one and incorrect answers resulted in a score of zero.

The test of statistical reasoning, on the other hand, is based on inductive reasoning and has also been previously used by Stanovich and West (1998) as stimulus material. The test was modified such that it would suite Scandinavian conditions. Hence, six decision scenarios were created where each task described a bipolar decision situation where one of the situations was supported by a statistical majority and the other was more individual and based on a subjective opinion. Each task included four rating scale options including the two end poles. Participants received scores depending on the extent to which their responses coincided with the statistical majority options. Four levels of scores were distributed (Alternative A, most certainly = 2; Alternative A, probably = 1.5; Alternative B, probably = 0.5; Alternative B, most certainly = 0) such that the highest levels represented the statistical majority options and the lowest denoted the individual and subjective options. The results from the two separate rationality tests were added in order to obtain a maximal total rationality score of 22 points (syllogisms: max 10 points; statistical reasoning: max 12 points) (*n* = 72, *M* = 13.96, *SD* = 3.12). 

### 2.7. Procedure

A series of pilot tests was conducted in order to create a booklet of reliable tests. These tests were subsequently conducted in two larger classes including undergraduate psychology students. The entire sessions lasted for about 30–40 min and specific time restrictions were imposed on the two introductory cognitive tests (cognitive style and analytical intelligence). The holistic independent test included five subtests that each had a time restriction set to two minutes. In the remaining three tests, (intuition, syllogisms, and statistical reasoning), no time restrictions were applied. Here, participants could use as much time as they wanted to fulfil the tasks. An experiment leader was always present during the sessions in order to assist and make oral clarifications. Before submission, all participants were instructed to check that all items had been properly filled in.

## 3. Results

Our first hypothesis implies that holistic independent individuals will be better rational performers than holistic dependent ones. This hypothesis was tested by using a one way ANOVA with holistic independence on framing (holistic dependent, holistic independent) as the independent variable and rationality scores as the dependent variable (*F* (1, 46) = 24.16, *p* < 0.001, *η*^2^ = 0.354). A reliable main effect was detected indicating support for Hypothesis 1 (Holistic dependent individuals *N* = 22; *M* = 12.10; *SD* = 6.05 Holistic independent individuals *N* = 24; *M* = 16.19; *SD* = 7.10).

Our second hypothesis states that holistic dependent individuals should use intuitive reasoning processes to a higher extent than holistic independent individuals. This hypothesis was tested with a one way ANOVA with holistic dependence/independence on framing (holistic dependent, holistic independent) as the independent variable and self-rated intuition as the dependent variable (*F* (1, 40) = 6.81, *p* < 0.10, *η*^2^ = 0.12). A marginally reliable main effect was observed in support of Hypothesis 2 (Holistic dependent individuals *N* = 15; *M* = 28.23; *SD* = 11.46 Holistic independent individuals *N* = 15; *M* = 24.72; *SD* = 9.23).

Our third hypothesis suggests that holistic dependence/independence on framing should better predict the dependent variables than analytical intelligence. In order to establish this, we first performed a correlation analysis of all the main variables (see Table 1).

Second, this hypothesis was tested with two step-wise multiple regression analyses (MRAs). In the first analysis, rationality was used as the criterion (dependent) variable and analytical intelligence and holistic dependence/independence on framing were applied as the independent variables, while controlling for age and gender (see Table 2). Here, two reliable predictors were found to better explain the variance observed in the dependent variable than analytical intelligence, namely holistic independence on framing (*β* = 0.56, *p* < 0.001) and age (*β* = 0.22, *p* < 0.05). Analytical intelligence only revealed a weak tendency to become significant (*β* = 0.17, *p* = 0.12). The collinearity between holistic dependence/independence on framing and analytical intelligence was tested as a manipulation check and revealed a correlation of *r* = 0.383, *p* < 0.001. This result was judged as satisfactory as the critical limit value is normally set to somewhere in between 0.60 and 0.70.

In the second stepwise multiple regression analysis we applied the same predictors but used self-rated intuition as the criterion (dependent) variable. Here, neither holistic dependence/independence on framing nor analytical intelligence was able to predict self-rated intuition reliably. (see Table 3). However, age did predict self-rated intuition (*β* = −0.36, *p* < 0.01).

### Gender as a Covariat to Cognitive Style

In an analysis of covariance (ANCOVA) gender was tested as a covariat to holistic dependence/independence on framing as the independent variable and rationality as the dependent one. Here, it was revealed that gender was a marginal explanator of the results revealed in connection with the test of Hypothesis 1 (*F* (1, 46) = 21.24, *p* < 0.001, *η*^2^ = 0.33).

## 4. Discussion

The main purpose of the present study was to investigate the importance of holistic dependence/independence on framing on rational performance and self-rated intuition. In this context, rationality is operationalized as as thinking and behaving in ways that maximise goal achievement based on available evidence (Stanovich & West, 1998, 2000). Another important aim was to clarify if holistic dependence/independence on framing would better predict rationality and intuitive processing than analytical intelligence. The results indicated a difference in how holistic dependent and holistic independent individuals perform rationally and intuitively. Holistic dependence/independence on framing was found to be a better predictor of rational performance than analytical intelligence. Additionally, holistic dependence/independence on framing showed a marginal tendency to predict self-rated intuition compared to analytical intelligence.

During the last decade, there have been numerous studies that have looked at the impact of thinking style and cognitive capacity on peoples’ reasoning and decision making (e.g., Stanovich & West, 1998, 2000). Most often, the need for cognition (NfC) has been chosen as the focal thinking style. However, the predictive power of NfC on peoples’ reasoning skills has been difficult to establish in these studies. The contribution of the present paper lies in the identification of holistic dependence/independence on framing as a quite good predictor of reasoning skills and decision making. Additionally, findings from previous research establishing strong correlations between holistic dependence/independence on framing and analytical intelligence (e.g., Linn & Kyllonen, 1981; López-Rupérez et al., 1991; Tsaparlis, 2005) have been replicated. Furthermore, we have extended this research through showing that holistic dependence/independence on framing seems to be related to both analytical and intuitive decision making in a rather systematic way.

Holistic dependence/independence on framing appears to be more strongly associated with reasoning skills and analytical decision making than intelligence in this sample. A parallel may be drawn to the area of inhibition. It has been shown lately that inhibitory capacity tests can be a better predictor of reasoning performance than more general ability tests (e.g., Markovits & Doyon, 2004). A clear-cut relationship between inhibition training and holistic dependence/independence on framing has also been observed (Moutier et al., 2002). Thus, inhibition may, like holistic dependence/independence on framing, include a broader range of complex capabilities in comparison to analytical intelligence. For instance, an interesting issue to consider could be how holistic dependence/independence on framing is connected to inhibition, since there are indeed many similar traits.

The obtained results were clearly in support of Hypothesis 1. Holistic independent individuals performed significantly better on the rationality tasks than holistic dependent individuals. In order to perform well on our rationality test (syllogisms and statistical reasoning), the ability to de-contextualize the logics and find the primary problems in the presented information may play a role (Stanovich & West, 1998). An interpretation of why holistic dependence/independence on framing correlates more with the rational thinking test than does the Raven test is that holistic dependence/independence on framing may share components with spatial ability. So, the correlation between holistic dependence/independence on framing and rational thinking could largely be mediated by a shared component of spatial ability (see also Chater, 1996, 1997, 1999; Chater & Vitanyi, 2003).

However, Hypothesis 2 was only marginally supported. Our results revealed that holistic dependence/independence on framing also leads to differences in self-rated intuition but the effect was found only to be marginally reliable. Here, holistic dependent individuals reported a higher degree of self-rated intuition than what holistic independent individuals did. However, this finding only revealed a very marginal significance. This tendency may therefore be regarded as a hypothesis which has not yet been falsified and would be a reasonable work hypothesis for future studies. An explanation of this tendency could be that holistic independent individuals tend to select more analytical decision strategies (lower scores on the intuition test) in order to deliberately assure themselves that they have chosen the best possible alternative. As holistic dependent individuals performed worse on the rationality test, it may be assumed that they prefer to apply their intuitive capabilities (higher score on the intuition test) rather than more analytically and reason-based approaches. Several studies confirm that holistic independent individuals are more task oriented, more attentive to detail, and more analytical than holistic dependent individuals (Fritz, 1994; Liu & Reed, 1994; Lyons-Lawrence, 1994; Reiff, 1996; Riding & Cheema, 1991). It may be suggested that holistic dependent and independent individuals differ in their use of intuition in relation to their rational and analytical capacity.

Finally, we received mixed support for Hypothesis 3. Holistic dependence/independence on framing was found to be more strongly associated with rational performance than analytical intelligence in this sample. This result may be interpreted such that holistic dependence/independence on framing includes a broader range of complex capabilities compared with analytical intelligence. This fact should explain the difference with regard to rational performance. There are several studies that support this interpretation revealing high degrees of correlation between cognitive style and several other cognitive abilities (Sternberg, 1997). However, our results showed that neither holistic dependence/independence on framing nor analytical intelligence was able to predict self-rated intuition. Nevertheless, a marginal ability was observed for holistic dependence/independence on framing. It is possible that this ability could have been verified if we had not operationalized at such a general level.

## 5. Limitations

Our test of holistic dependence/independence on framing had very high reliability, with a good distribution. Nevertheless, our time pressure and scoring procedures may be questioned, since they have not been tried before. Still, the results, in this context, seem to indicate a good distinction between participants’ performances. The test of analytical intelligence also received high reliability scores with a good distribution. We decided to apply only one test of analytical intelligence in order to better be able to focus on the relationship between holistic dependence/independence on framing and analytical intelligence. Another reason was to limit the use of tests in the entire study. Given this, we only used the instrument in order to measure the analytical part of intelligence, which is part of general intelligence.

With regard to our intuition test, it must be revealed that the concept was difficult to operationalize, since intuition is built on individual experiences and is not open for introspection. For this reason, it became too complicated to test intuitive performance. Instead, we tested participants’ attitudes toward using their intuition in a couple of scenarios. Through this, we obtained indirect measures on their use of intuition. Focusing on the test of rational performance, we relied to a great extent on material developed by Stanovich and West (1998). Since this test material has been used to a large degree by other scientists, and is widely cited, reliability has been externally established and recognised. Consequently, we believe that we can rely on the two tests of theirs that have been applied in our study. It is important to note, however, that our measure of rationality only rests on syllogisms and statistical reasoning. We would have optimally liked to have applied more dimensions of rationality, in line with Stanovich and West. Nevertheless, in order to limit our general number of tests, we believed it to be satisfactory to test inductive and deductive reasoning (statistical reasoning vs. syllogisms) as a means to measure rational performance in a reliable way.

Another issue is that we only used a limited section of a full scale IQ test and that the validity of this section only has only been partially established (Ravens matrices). For this reason, it may be argued that the criticism directed towards IQ tests lacks validity. However, it must be noted that Ravens’ matrices have been used extensively in practical settings as a useful assessment tool for IQ. The Progressive Matrices have been described as one of the purest and best measures of *g* or general intellectual functioning, available.

Finally, it must be observed that the present study is purely correlational and do not provide any experimental support. For instance, the original Stanovich and West studies have recently been followed-up by secondary task studies that showed that a lack of executive resources is directly causing faulty analytic reasoning and decision making (de Neys, 2006).

### 5.1. Future Research

A great challenge lies in trying to establish the effect of holistic dependence/independence on framing on intuitive decision making. The present results only give an indication of a partial connection, and the scale measuring self-reported intuition has not yet been thoroughly validated. If this connection could be more fully established it would give further support for the notion that the availability heuristic (Kahneman, 2003; Selart et al., 2006) plays a major part in explaining the influence of holistic dependence/independence on framing on analytical and intuitive decision making.

It would also be interesting to study the role of syllogisms more in detail. An important division may be made between abstract syllogisms and syllogisms with a belief-logic conflict. Stanovich and West have used both types in their studies. Focusing on belief-laden syllogisms, it would be perfectly possible to partial out the effects of logic and believability on two separate indices (e.g., see Newstead et al., 2004). In such a case, holistic independent individuals should score higher on the logical index whereas holistic dependent individuals should score higher on the belief-based index. Such results would in many ways clarify the relation between holistic independence and inhibition.

Another interesting line of research would be to contrast holistic dependence/independence on framing, treated as a cognitive style, with contextual framing effects that have a bearing on emotions. For instance, it was found by Gross and D’Ambrosio (2004) that different frames can influence emotional responses to political events, suggesting that frames alter the relationship between individual predispositions and emotional responses. In line, Druckman and McDermott (2008) showed how emotions and framing effects interplay in risky decision-making. Gross (2008) similarly found that emotional responses have the ability to mediate the effects of framing on policy opinions, suggesting that both affective and cognitive processes are at play in shaping public attitudes. It was also revealed by Keller et al. (2003) how a recipient’s affective state (positive or negative mood) interact with the framing of loss vs. gain messages. Finally, Giorgi (2017) launched a new model that can predict and analyse frame effectiveness, revealing the interplay between cognition and emotion among audiences.

### 5.2. Implications for Practice

The findings in this study have several possible implications for practice. Organizations seek to hire and promote individuals with solid analytical decision making skills (Hunt et al., 1989; Chan, 1996; Graff, 2003; Sadler-Smith & Smith, 2004; Kozhevnikov, 2007; Glambek et al., 2026). Some companies explicitly test for reasoning abilities or even intelligence. This study shows that holistic dependence/independence on framing does explain a sizable portion of the variation in performance on analytical skills. Moreover, we have demonstrated that holistic dependence/independence on framing shows discriminative validity in relation to intelligence. However, intelligence testing has been criticized for assessing limited and in some cases irrelevant skills when applied to real life contexts. Other criticisms are that intelligence tests are too elementary and fail to capture cognitive creative problem solving capabilities by asking people to choose one correct answer (Menkes, 2005).

In contrast to intelligence tests, tests of holistic dependence/independence on framing provide an indication of peoples’ ability to de-contextualize; an ability that is immediately useful in real life, as people in everyday life are forced to absorb and digest vast amounts of information. How people handle information and their ability to apply analytical thinking in situations where there exist an abundance of (sometimes) superfluous information is most likely highly relevant to how an employee performs in his or her job. For instance, it has been shown by Hodgkinson et al. (1999) that the framing bias is likely to be an important factor in judgment and decision making and the ability to work with cognitive mapping provides an effective means of limiting the damage accruing from this bias.

A second implication is that people depending on their holistic dependence/independence on framing seem to apply different strategies of decision making. If holistic dependent individuals apply different tactics and strategies from holistic independent individuals, this knowledge would be useful for organizations to better assist employees in improving on their analytical and decision making skills. Holistic dependent individuals may through training become better at de-contextualizing information and thus become better at their decision making skills, or they may, by becoming aware of their cognitive style, develop meta-skills by which they are able to monitor and choose optimal strategies based on their cognitive style. According to Klein (1998), it is important that people in organizations understand that the perspective that they employ provides a set of values and salient issues that influence the factors that we consider in our decision-making processes. As a result, it influences our final choice. Holistic dependent decision-makers train themselves in developing a full understanding of the situation by explicitly viewing decision problems from divergent perspectives. Such different frames may, for instance, include an engineering/technology frame, a sales/marketing frame, a production frame, a political frame, a legal frame, an accounting/finance frame, a competitive frame, and an ethical frame.

Finally, there may be tasks and areas in which holistic dependents excel. The creativity research literature thus emphasizes the role of intuition in producing novel and creative ideas (Ho & Kozhevnikov, 2023; Gu et al., 2025; Kokorina & Sementsova, 2025; Zivi et al., 2025). Highly creative people have been found to be highly receptive to contextual cues just like affective dependent individuals. Testing people for holistic dependence-independence then may be used to place people in areas in which these specific skills are likely to be particularly useful and in which the possible drawbacks from holistic dependence are less prominent (Glambek et al., 2026). Rather than seeing either analytical or intuitive strategies as superior to each other, optimal strategies may combine these strategies and apply them to different stages in a decision making process (Strack & Deutsch, 2004). Nevertheless, how such combinations are achieved will depend on how holistic dependence/independence on framing is perceived. If holistic dependence/independence on framing is found to be a fixed personality trait, combinations will have to be achieved through the composition of work groups. If holistic dependence/independence on framing can be taught or influenced, individuals may be able to adapt their holistic dependence/independence on framing to different problems.

## 6. Conclusions

A major aim of the present study was to investigate what implications cognitive style shaped by framing has on rational and intuitive thinking. A booklet of relevant tests was distributed to university students who served as participants. It was found that participants with a high holistic independence on framing scored higher on rational thinking tasks than those who scored low. High holistic independence on framing was also found to be a better predictor of rational thinking than was analytical intelligence. The results are discussed and related to the more general issue of how cognitive style may impact upon rational and intuitive thinking.

## Figures and Tables

**Table 1:** A correlational analysis (Pearson) of the main variables (*N* = 72).

Variables	1	2	3	4
1. Cognitive Style	-	0.383 **	−0.130	0.555 **
2. Intelligence		-	0.019	0.360 **
3. Intuition			-	−0.204
4. Analytical decision making				-

** significant, *p* < 0.01.

**Table 2:** A multiple step-wise regression analysis based on predictors of rationality (*n* = 67).

Variables	B	β	*p*
Step 1			
Cognitive style	0.21 (0.04)	0.56	<0.001
Excluded Variables			
Analytical Intelligence		0.17	0.12
Gender		−0.07	0.53
Age		0.22	0.04
Step 2			
Cognitive style	0.23 (0.04)	0.59	<0.001
Age	0.17 (0.08)	0.22	0.04
Excluded Variables			
Analytical Intelligence		0.18	0.09
Gender		−0.06	0.59

Note: B is a non-standardized regression coefficient with the standard error in parentheses; β is the standardized regression coefficient; R = 0.56, R^2^ = 0.31, F = 29.45, *p* < 0.001 for Step 1; R = 0.60, R^2^ = 0.35, F = 17.81, *p* < 0.001 for Step 2.

**Table 3:** A multiple step-wise regression analysis based on predictors of self-rated intuition (*n* = 49).

Variables	B	β	*p*
Step 1			
Age	−0.48 (0.18)	−0.36	0.01
Excluded Variables			
Cognitive style		−0.17	0.15
Analytical Intelligence		0.01	0.96
Gender		0.22	0.36

Note: B is a non-standardized regression coefficient with the standard error in parentheses; β is the standardized regression coefficient; R = 0.36, R^2^ = 0.13, F = 7.20, *p* = 0.01 for Step 1.

## Data Availability

The data presented in this study are available on request due to ethical restrictions.
